# Novel heterozygous c.798C>G and c.1040T>G mutations in the *GBA1* gene are associated with a severe phenotype of Gaucher disease type 1

**DOI:** 10.1007/s00277-014-2036-x

**Published:** 2014-02-28

**Authors:** Maciej Machaczka, Monika Klimkowska

**Affiliations:** 1 Hematology Center Karolinska, M54, Karolinska University Hospital Huddinge, 141 86 Stockholm, Sweden; 2 Medical Faculty, University of Rzeszow, Rzeszow, Poland; 3 Department of Clinical Pathology and Cytology, Karolinska University Hospital Huddinge, Stockholm, Sweden

Dear Editor,

Gaucher disease (GD) is a progressive, multisystem, autosomal recessive lysosomal storage disorder caused by the deficient activity of the lysosomal enzyme, glucocerebrosidase, arising from mutations in the *GBA1* gene (1q21) [1]. Hematological symptoms, such as thrombocytopenia or splenomegaly, are frequent disclosing signs of GD type 1 (GD1).

Here, we report the case of a young Iranian woman, originating from the city of Ahvaz in the southwest Iran (Khuzestan Province) but living in Sweden since the age of 16 years. The ancestors of her both parents migrated to Iran from Caucasus region in Georgia. Although the parents of the patient had a consanguineous relationship (cousins), they were not aware of any previous cases of GD in their family, apart from their own children.

**Fig. 1:**
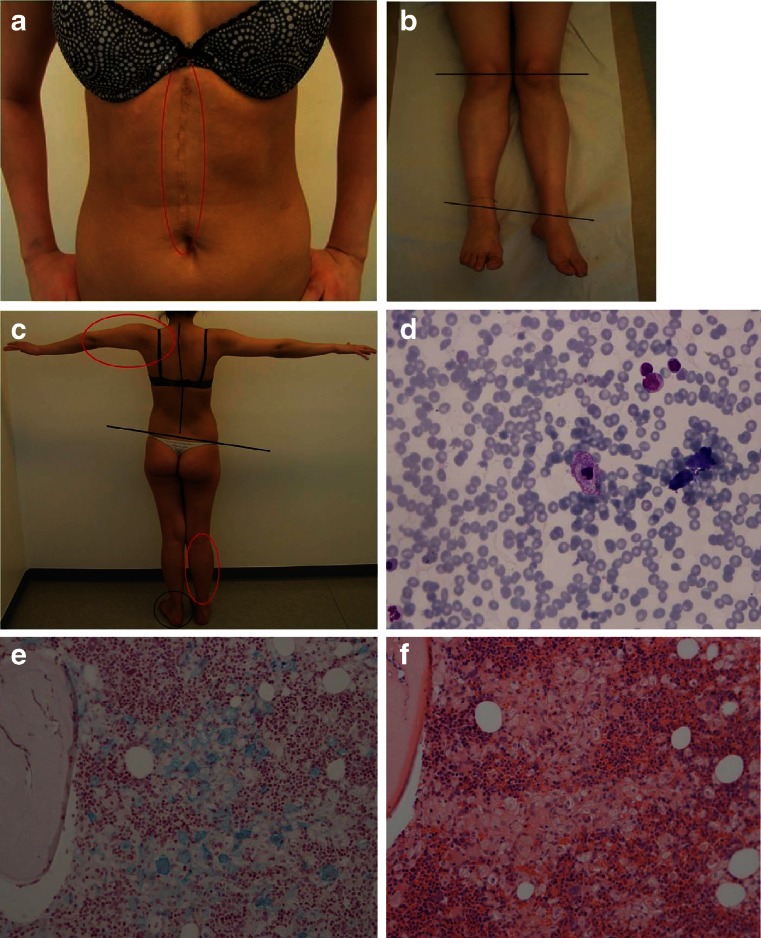
**a** A large scar after splenectomy. **b**, **c** Primary skeletal deformations of the right leg and the left arm (*marked red*), followed by the secondary deformations of the spine and the left feet (*marked black*). **d** Bone marrow cytology; centrally placed histiocyte with Gaucher cell morphology (MGG stain). **e**, **f** Bone marrow histology; prominent histiocytic infiltrates composed of Gaucher cells; iron-hematoxylin stain (**e**) and HE stain (**f**)

At the age of 4, the patient developed a huge belly, “as a pregnant woman” according to the mother’s relation. She was splenectomized 1 year later due to massive splenomegaly and increased bleeding tendency (Fig. 1a). Histopathology of the spleen disclosed the presence of storage histiocytes with Gaucher cell morphology. However, further enzyme assay or *GBA1* mutation analysis was not performed at that time. Later on, the patient suffered periodically from a severe skeletal pain, followed by the skeletal deformations in her right leg and left arm (Fig. 1b, c).

Noteworthy, the patient had four older siblings. Two oldest brothers are healthy and alive. The third brother, 1 year older than the patient, died at the age of 5 years, having at that time a huge belly. He was never splenectomized. Postmortem examination revealed histopathology of GD. Her 6 years older sister was splenectomized at the age of 5 due to massive splenomegaly and easy bruising. The spleen histopathology disclosed a morphological picture of GD. After some time, she started to experience severe skeletal pain accompanied by increasing hepatomegaly and bleeding problems. The patient’s sister died at the age of 21 years, having a large abdomen “as during late pregnancy,” due to a massive hepatomegaly, according to the patient’s relation. None of the affected siblings, including the patient, displayed any neurologic symptoms. None of them was treated in Iran with enzyme replacement therapy (ERT) either.

The patient’s mother, 1 year after the death of her second child because of GD, decided to immigrate with her last daughter to Norway, in hope to allow the patient ERT and to avoid the fate of older siblings with GD. However, the patient did not start ERT in Norway, and she moved with her mother to Sweden.

The final diagnosis of GD was confirmed in Sweden by disclosing Gaucher cells in the bone marrow (Fig. 1d–f) and then reduced activity of glucocerebrosidase in peripheral blood leukocytes (0.49 μkat/kg protein; normal range 2.1–3.8) and increased activity of plasma chitotriosidase (9,743 nkat/L; normal range <40). Further direct DNA sequencing of the coding exons and intron/exon borders in the *GBA1* gene revealed the heterozygous mutations c.798C>G and c.1040T>G.

The mutation c.798C>G results in amino acid change from phenylalanine to leucine at position 266 (p.Phe266Leu), which has slightly different physicochemical properties than phenylalanine. The mutation c.1040T>G results in the change of the amino acid isoleucine at position 347 into serine (pIle347Ser). There is a large physicochemical difference between Ile and Ser. Both phenylalanine at position 266 and isoleucine at position 347 are highly conserved between species (up to *Caenorhabditis elegans*). To the best of our knowledge, both aforementioned mutations in the *GBA1* were never before reported in patients with GD.

The patient started ERT with imiglucerase (Cerezyme™) at the dose of 60 IU/kg at the age of 17 years. With time, she responded very well to the ERT, experiencing normalization of the liver size and her body weight as well as normalization of the whole blood hemoglobin concentration and platelet count. She has never again experienced acute skeletal pain. Nevertheless, she experiences from time to time pain related to her persistent skeletal deformities. When on ERT, she was able to begin studies, gain a profession, and find a job. Currently, the patient is almost 26 years old and clinically stable. Thanks to ERT, which she has been receiving for 9 years now, she lives an almost normal life.

The onset of GD in early childhood, an aggressive course in respect to visceral, hematologic, and skeletal domains of GD, as well as the lack of neurologic symptoms indicate a severe form of GD1 in the reported patient and her two deceased siblings. Importantly, the present case illustrates a striking ability of ERT to reverse an uniformly lethal outcome of GD caused by the novel heterozygous mutations c.798C>G/c.1040T>G in the *GBA1*.

Although GD is particularly prevalent among Ashkenazi Jews, it can be found in all ethnic groups [1]. The clinical presentation of GD is highly variable [2–4], and in the absence of a known affected family member, GD1 remains a diagnostic challenge and is often not included in differential diagnosis of thrombocytopenia even by experienced hematologists [5]. A suspicion of GD based on morphological findings, such as the presence of Gaucher cells in any tissue sample, should always be confirmed by enzymatic and genetic assays [1]. Finally, this family story is an obvious reminder that ERT does save lives in GD.
